# Supplementary material for: Hybrid Models and Biological Model Reduction with PyDSTool
Source: PLoS Comput Biol. 2012 Aug 9;8(8):e1002628. doi: 10.1371/journal.pcbi.1002628 (PMC3415397; doi:10.1371/journal.pcbi.1002628)
Supplement: Text S4 — Complete source code for the PyDSTool package (version 0.88.120504). Includes API documentation and help files linking to web pages. This file is identical to the current public release on Sourceforge.net. (ZIP) [file pcbi.1002628.s004.zip › PyDSTool/html/toc-PyDSTool.fixedpickle-module.html]

xml version="1.0" encoding="ascii"?


fixedpickle


# Module fixedpickle

---

## Classes

PickleError  
 Pickler  
 PicklingError  
 Unpickler  
 UnpicklingError  

\_EmptyClass

\_Stop

## Functions

\_keep\_alive

\_test

decode\_long

dump  
 dumps  

encode\_long

load  
 loads  

whichmodule

## Variables

APPEND  
 APPENDS  
 BINFLOAT  
 BINGET  
 BININT  
 BININT1  
 BININT2  
 BINPERSID  
 BINPUT  
 BINSTRING  
 BINUNICODE  
 BUILD  
 DICT  
 DUP  
 EMPTY\_DICT  
 EMPTY\_LIST  
 EMPTY\_TUPLE  
 EXT1  
 EXT2  
 EXT4  
 FALSE  
 FLOAT  
 GET  
 GLOBAL  
 HIGHEST\_PROTOCOL  
 INST  
 INT  
 LIST  
 LONG  
 LONG1  
 LONG4  
 LONG\_BINGET  
 LONG\_BINPUT  
 MARK  
 NEWFALSE  
 NEWOBJ  
 NEWTRUE  
 NONE  
 OBJ  
 PERSID  
 POP  
 POP\_MARK  
 PROTO  
 PUT  

PyStringMap

REDUCE  
 SETITEM  
 SETITEMS  
 SHORT\_BINSTRING  
 STOP  
 STRING  
 TRUE  
 TUPLE  
 TUPLE1  
 TUPLE2  
 TUPLE3  
 UNICODE  

\_tuplesize2code

classmap

compatible\_formats

format\_version

---

[hide private]
